# Breast implant associated anaplastic large cell lymphoma (BIA-ALCL): a challenging cytological diagnosis with hybrid PET/MRI staging and follow-up

**DOI:** 10.1007/s12282-020-01178-w

**Published:** 2020-11-01

**Authors:** Francesco Verde, Elena Vigliar, Valeria Romeo, Maria Raffaela Campanino, Antonello Accurso, Luigi Canta, Nunzia Garbino, Luca Basso, Carlo Cavaliere, Emanuele Nicolai, Massimo Imbriaco

**Affiliations:** 1 Department of Advanced Biomedical Sciences, University of Naples “Federico II”, Via S. Pansini, 5, 80131 Naples, Italy; 2 Department of Public Health, University of Naples “Federico II”, Via S. Pansini, 5, 80131 Naples, Italy; 3 Department of Clinical Medicine and Surgery, University of Naples Federico II, Via S. Pansini 5, 80131 Naples, Italy; 4 Unit of Plastic and Reconstructive Surgery, AOU Federico II, Via S. Pansini, 5, 80131 Naples, Italy; 5 IRCCS SDN, Via Emanuele Gianturco 113, 80143 Naples, Italy

**Keywords:** BIA-ALCL, Peri-implant effusion, Breast cancer, PET/MRI

## Abstract

We report a case of a 55-year-old woman with left breast cosmetic augmentation performed 5 years earlier, showing at ultrasound a left small amount of peri-implant effusion suspicious for an anaplastic large cell lymphoma localization. The final diagnosis was obtained by cytology using a small amount of fluid (6 ml). Subsequently, hybrid 18F-FDG PET/MRI was used for pre-operative staging and follow-up. An appropriate management of BIA-ALCL could be obtained even in cases of a small amount of peri-implant effusion, using a comprehensive approach of clinical and imaging evaluation, including PET/MRI as useful and innovative staging imaging technique.

## Introduction

Peri-implant breast seroma is a common complication of augmentation mammoplasty and breast prosthetic reconstruction, which clinically appears as a notable breast swelling, asymmetry or with breast pain [1]. Usually, it develops a few weeks or months after the breast implant surgery, whereas its occurrence in the late postoperative period (i.e. > 12 months) is very rare [2]. It has been suggested that the pathophysiology of late-onset periprosthetic effusion development may be related either to an inflammatory response to a bacterial infection, mechanical forces from traumatic injuries (e.g. haemorrhage, hematoma), or to malignant effusion due to a primary breast cancer or to breast implant-associated anaplastic large cell lymphoma (BIA-ALCL) [3]. Timely diagnosis of BIA-ALCL in women with late peri-implant effusions is critical as most cases of BIA-ALCL manifest as delayed seromas [4].

BIA-ALCL is a rare primary non-Hodgkin T-cell lymphoma and it has been recently included within the group of anaplastic lymphoma kinase (ALK) negative ALCLs [5]. It could arise as a solid mass attached to the prosthetic capsule and soft tissue or, more frequently, as a late peri-implant seroma within which tumour cells are enclosed [6]. As previously shown by Quesada et al. [7], the peri-implant effusion developing in BIA-ALCL does not correspond actually to a seroma, since it is composed of dense liquid derived from necrotic tumoral cells.

We hereby describe a rare case of a woman evaluated in our institution for a late breast implant effusion representing the only sign of BIA-ALCL, with the final diagnosis reached by a cytological sampling of a small amount of fluid; moreover, this case highlights the role of advanced imaging techniques such as hybrid ^18^F-FDG PET/MRI, in the diagnostic workup of this rare condition.

## Case report

A 55-year-old woman with left breast cosmetic augmentation presented at our institution complaining of left breast tenderness and mild swelling. The patient underwent left breast augmentation with retro-glandular implantation of a textured silicone prostheses 5 years earlier. Physical examination showed a mildly swollen and tense left breast, without any palpable axillary lymphadenopathy. According to the 2019 National Comprehensive Cancer Network (NCCN) guidelines on diagnosis and treatment of BIA-ALCL, a preliminary ultrasound (US) examination of the left breast was performed, revealing the presence of a small peri-implant fluid collection and a normal implant without any signs of capsular rupture.

Because of the absence of any traumatic or infectious causes, the patient underwent an US-guided fine-needle aspiration (FNA) and, given the small amount of peri prosthesis effusion visible on US, only 6 ml of cloud, yellowish fluid, was collected. The sample was concentrated by centrifugation; cytospins were prepared and Papanicolaou stained. Subsequently, a cell block (CB) was prepared from residual material to perform ancillary techniques.

**Fig. 1:**
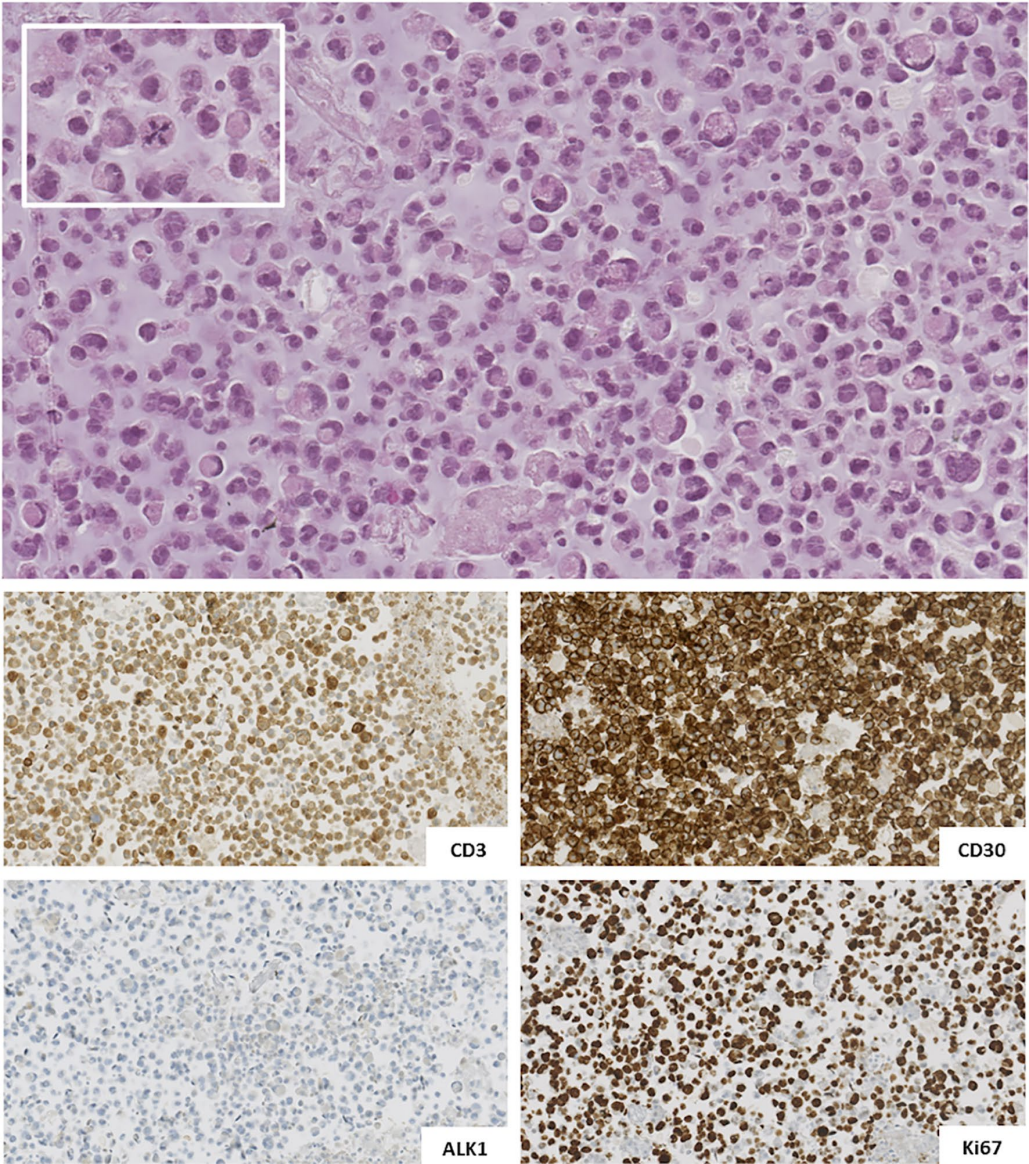
Cytophatological features of breast implant-associated anaplastic large cell lymphoma (BIA-ALCL) in peri-prosthesis effusion aspirates: hematoxylin–eosin stained CB slide showed a high cellular sample composed by medium to large-sized discohesive atypical cells with irregularly-shaped, hyperchromatic nuclei; larger cells showed peripherally-located, “horseshoe” shape nuclei and abundant clear cytoplasm. Atypical mitoses were also observed (inset) (**a**). Immunohistochemical studies on cell block material from the peri-implant fluid collection showed diffuse CD3 and, CD30 positivity and ALK1 negativity in atypical cells; Ki-67 labeling index was > 80% (**b**)

Papanicolaou stained cytospin preparations and hematoxylin–eosin stained cell blocks showed a high cellular sample composed by medium to large-sized atypical cells with irregularly-shaped, hyperchromatic nuclei. Of note, larger cells showed peripherally-located, horseshoe shape nuclei and abundant clear cytoplasm. Apoptotic cells and atypical mitoses were also observed. Immunohistochemical (IHC) evaluation showed a T-cell profile comprising of diffuse CD3 positivity, diffuse CD30 positivity and ALK1 negativity in atypical cells; Ki-67 labeling index was > 80% (Fig. 1).

**Fig. 2:**
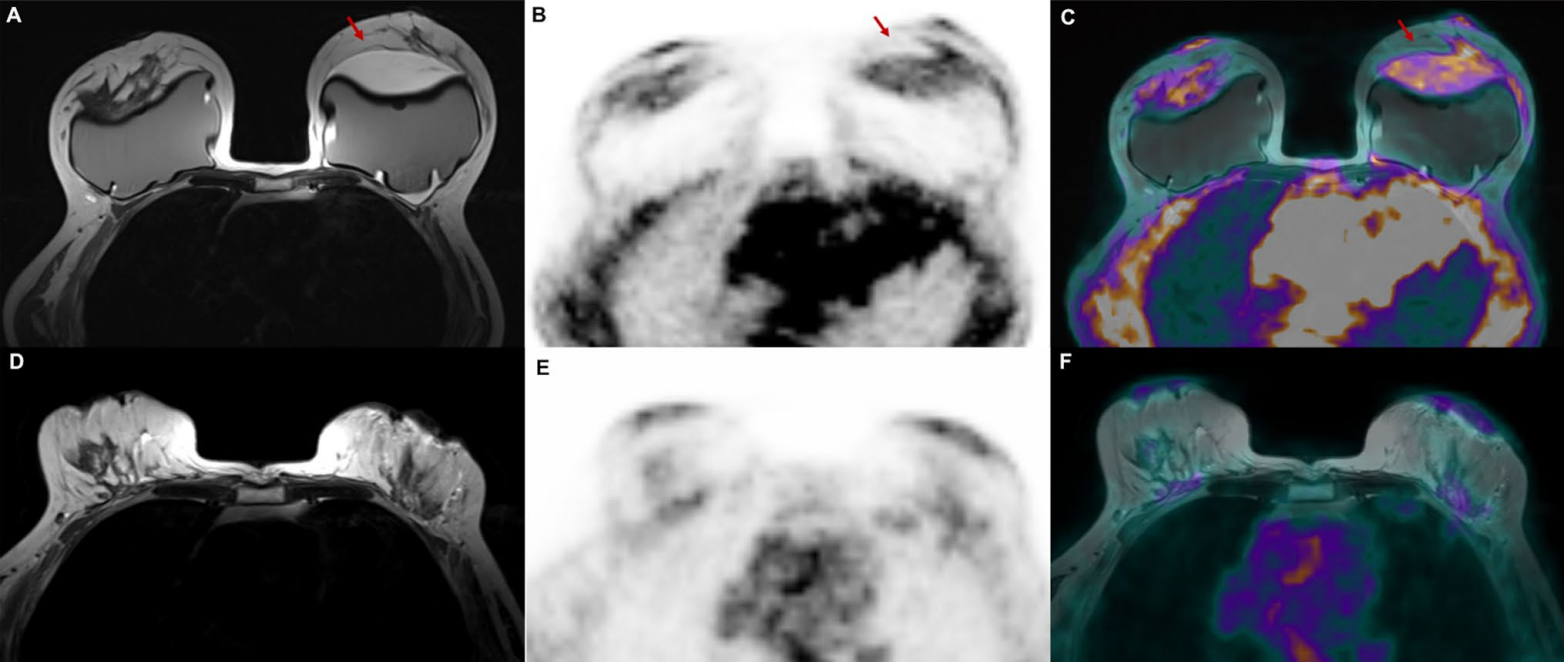
T2-weighted MR axial images (**a**, **d**). PET axial images (**b**, **e**). PET/MRI axial fused images (**c**, **f**). **a–c** A moderate fluid collection is appreciable around the left breast implant (**a**, arrow). The fluid collection also showed mild 18F-FDG uptake (**b**, **c** arrow). **d–f** Post-surgical follow-up examination performed after bilateral implant and capsule removal. No significant tracer uptake nor pathological enhancement over breast tissue are detectable

**Fig. 3:**
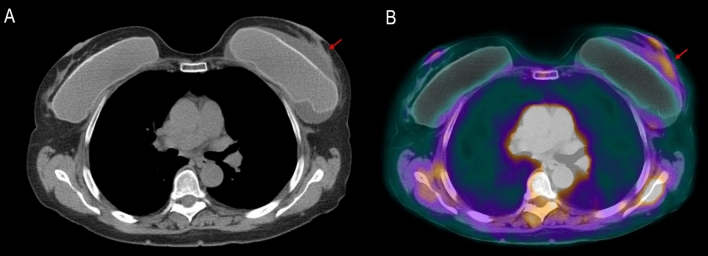
Axial unenhanced PET/CT image shows a small fluid collection surrounding the left breast implant (**a**, arrow) with mild tracer uptake on PET/CT fused image (**b**, arrow)

After the BIA-ALCL diagnosis was established, based on cytological and IHC characteristics of the FNA fluid sample, a pre-operative simultaneous ^18^F-FDG PET/MRI of the breast and the whole body using a 3 T Biograph mMR (Siemens Healthcare, Erlangen, Germany) was performed. MR axial T2-weighted images showed a moderate fluid collection around the left breast implant, higher than those seen on US, with mild tracer uptake (Fig. 2a–c) on PET images. No areas of increasing tracer uptake or of abnormal enhancement were detected beyond the peri-implant effusion neither within the breast tissue and no pathological axillary lymph-nodes were found. Prior to PET/MRI, unenhanced PET/CT scan was also performed showing a small volume effusion surrounding the left breast implant with mild tracer uptake on PET/CT fused images (Fig. 3a, b). Patient’s clinical-pathological features and imaging findings are reported in Table 1.

**Table 1:** Patient’s clinical-pathological features and imaging findings

Clinical features
Age: 55 years
Reason for implant: cosmetic
Implant side and location: left retroglandular
Time interval between implant and late seroma: 28 months
Content of implant: silicone
Surface of implant: textured
Physical exam: mild swelling
Pathological features
FNA fluid collection sample volume: 6 ml
Cytology: atypical cells with abundant cytoplasm and irregularly-shaped hyperchromatic nuclei
IHC: CD3 + , CD30 + , ALK1-, Ki-67 labeling index > 80%
Imaging findings
US findings: scant anechoic pericapsular fluid collection in the left breast
PET/CT findings: small volume effusion around the left breast implant with mild tracer uptake on PET images
PET/MRI findings: moderate fluid collection around the left breast implant showing a mild 18F-FDG uptake and no areas of abnormal enhancement were detected at the level of the breast parenchyma

The patient underwent bilateral implant and capsule removal and subsequent histological control confirmed BIA-ALCL diagnosis (Table 1), which was confined to the capsule. Follow-up ^18^F-FDG PET/MRI of the breast and the whole-body scan, performed at 1-month showed no tracer uptake nor areas of abnormal enhancement (Fig. 2d–f). The patient did not undergo chemotherapy nor radiotherapy and she is currently on clinical follow-up.

## Discussion

We report a case of a 55-year-old woman with left breast cosmetic augmentation performed 5 years previously, showing a left small peri-implant effusion that turned out to be the site of ALCL. In particular, the diagnosis was obtained by cytology using a small amount of fluid (6 ml) and advanced imaging techniques i.e. hybrid PET/MRI were used for pre-operative staging and follow-up.

BIA-ALCL is a rare T-cell lymphoma, which commonly manifests as a peri-implant effusion occurring at least 1 year following cosmetic or reconstructive breast implantation with a median time from implantation to the diagnosis of 8 years [8]. In 2016, the World Health Organization classified BIA-ALCL as a distinct lymphoma from primary breast lymphoma and, in the same year, the NCCN released evidence-based consensus guidelines for the diagnosis and treatment of the disease which have been updated in 2019 [9]. In one-third of cases, BIA-ALCL presents as a solid mass infiltrating the prosthetic capsule which could represent a successive stage of the disease [10]. Textured implants are most commonly associated with the incidence of BIA-ALCL whereas no difference has been observed for location (subpectoral or retro-glandular) and content (saline or silicone) of the used devices [11]. Among multiple possible pathogenesis theories, recent studies have demonstrated that chronic inflammation caused by textured implants may act as a trigger of T-cell response that subsequently develops into an ALCL [2].

Patients usually present with breast swelling, asymmetry and tenderness and the clinical evaluation tends to consider suspicious for BIA-ALCL any collection appearing greater than 1 year after implantation and not associated with traumatic or infectious causes.

Given the excellent prognosis of the localized early stage of BIA-ALCL [1], a prompt imaging recognition of suspect late peri-implant seroma is crucial. Breast US is the first diagnostic test to assess the presence and the extent of peri-implant effusion and to evaluate any associated capsule masses or swollen regional lymph nodes; indeed, an integrated evaluation of regional lymphadenopathy is recommended as almost 20% of patients could exhibit associated axillary lymphadenopathy as demonstrated by Ferrufino et al. [12]. In this regard, a previous work by Aladily et al. [13] observed that patients with BIA-ALCL mass pattern experienced a worse disease course, including regional lymph node involvement.

MRI is adopted for US equivocal cases, allowing to accurately evaluate other breast implants complications, such as implant ruptures as a cause of peri-prosthetic fluid collection [14, 15]. Peri-implant effusion typically appears a hyperintense fluid collection around the implant on T2-weighted sequence [16]. In a previous work, Adrada et al. [17] evaluated the sensitivity and specificity of different imaging methods for recognize BIA-ALCL and they found that US and MRI were the most performing imaging methods for detecting peri‐implant collections, with 84% and 82% sensitivity, while PET/CT achieved the highest sensibility (64%) in assessing the solid mass pattern of BIA-ALCL.

The cytomorphological and IHC features of the cellular composition in the effusion fluid are crucial in BIA-ALCL diagnosis. Therefore, US-guided FNA is performed to sample the peri-prosthetic collection and, according to the NCCN BIA-ALCL guidelines, as much fluid as possible should be collected (minimum 50 ml) to provide enough material to cytological examination. Regarding US aspiration technique, it is important to correctly position the patient to localize the fluid collection in its most dependent position [18]. Furthermore, for cases with small effusions, it is necessary to apply light pressure to expand the target window for aspiration [19]. BIA-ALCL is characterized by the presence of the “hallmark cells”, large lymphoid cells with abundant cytoplasm and horseshoe-shaped nuclei. Immunophenotypically, all tumour cells are positive for CD30 and negative for ALK and show variable expression of one or more T cell markers, such as CD3 and CD4 [20]. BIA-ALCL presenting with minimal peri-implant effusion represent a very diagnostic challenge, as described in a prior work by Miranda et al. [21], which discovered incidentally BIA-ALCL by a small amount of effusion in patients who had surgery for other reasons or in cases undergoing contralateral breast implant removal. In our case, appropriate management of cytological samples was obtained providing a comprehensive morphological and IHC evaluation, even if a scant peri-implant fluid was collected through US-guided FNA.

For any confirmed cases of BIA-ALCL, the 2019 NCCN guidelines suggest performing preoperative PET/CT to assess the presence of associated capsular masses, regional involvement or lymphadenopathy [9]. In our case, we evaluated oncological staging with PET/MRI, simultaneously obtaining morphologic, metabolic and functional parameters. Furthermore, MR T2-weighted images allowed us to assess and quantify the amount of peri-prosthesis effusion more accurately than US evaluation. We think the difference could be explained for the different positioning of the breast, resulting prone in PET/MRI and supine in US. Similarly, the left peri-implant fluid collection was better depicted on PET/MRI as compared to PET/CT images, due to: (1) the superior resolution of MRI in breast tissue and fluid collection; (2) the prone position of the patient on MRI that facilitated the collection of the fluid anteriorly to the prosthesis.

Hybrid PET/MRI is an emerging and promising imaging technique especially for oncological application providing all the parameters that could be gathered from MRI and PET examinations, being applied in different clinical settings, from staging to the assessment of the response to neoadjuvant chemotherapy [22–24]. In this regard, the oncological workup of BIA-ALCL could benefit from the use of PET/MRI for the high contrast resolution of MRI in breast tissue and for quantitative data derived from PET, DWI, and perfusion MRI which offer more diagnostic tools in the evaluation of tumour extension, nodal involvement and for the detection of distant metastasis.

To date, this is the first case report exploiting PET/MRI technique for pre-operative staging and follow-up of BIA-ALCL. Although PET/MRI is not a widespread imaging method, it has shown important advantages like lower radiation dose in comparison to PET/CT in case of whole-body staging or post-treatment surveillance [25]. Furthermore, PET/MRI could provide an amount of novel imaging features which may eventually be converted for radiomic analysis enabling a personalized diagnostic and therapeutic pathway [26].

## Conclusion

In conclusion, appropriate management of late seromas (> 1 year), consisting of an integrated approach of clinical and imaging evaluation, US-FNA, cytological and IHC studies should be performed even in cases of a small amount of peri-implant effusion. Furthermore, PET/MRI can provide comprehensive morpho-functional imaging information which improves patients’ management in this newly recognized sub-type of lymphoma.
